# Description and Analysis of a Novel Subtype of the Anti-Synthetase Syndrome Characterized by Frequent Attacks of Fever and Systemic Inflammation in a Single-Center Cohort Study

**DOI:** 10.3389/fimmu.2021.729602

**Published:** 2021-09-23

**Authors:** Shuhui Sun, Zhiwei Chen, Danting Zhang, Wenwen Xu, Wanlong Wu, Fangfang Sun, Liyang Gu, Jie Chen, Jiajie Li, Ting Li, Xiaodong Wang, Shuang Ye

**Affiliations:** Department of Rheumatology, Renji Hospital, Shanghai Jiaotong University School of Medicine, Shanghai, China

**Keywords:** idiopathic inflammatory myopathies, anti-synthetase syndrome, fever, inflammation, anti-PL-7

## Abstract

**Objectives:**

The aim of this study was to investigate anti-synthetase syndrome (ASyS) patients who presented with recurrent episodes of fever and systemic inflammation.

**Methods:**

A retrospective cohort of Chinese ASyS patients (n=126) in our center (between January 2013 and January 2020) was included. Patients presenting with concomitant autoimmune rheumatic diseases or malignancies were subsequently excluded. The number of non-infectious fever attacks and attack frequency were recorded and calculated. Patients with two or more attacks and within the upper three quartiles of attack frequency were defined as high-inflammation group. Univariate and multivariate analyses were carried out to characterize the high-inflammation subtype.

**Results:**

Out of 113 eligible patients with an average of 5 years follow up, 25 patients were defined as the high-inflammation group (16 for anti-Jo1, 9 for anti-PL7), with an average of 1.12 attack/patient-year. Compared to low-inflammation group (0–1 attack only and a frequency lower than 0.5 attack/patient-year), the high-inflammation group had higher occurrence of fever and rapid progressive interstitial lung disease (RPILD) as the first presentation (84% *vs.* 21% and 40% *vs.* 9%, respectively, both p<0.01). Anti-PL-7 was related to the more inflammatory phenotype (p=0.014). Cumulative disease-modifying agent exposures (>=3) were much higher in the high-inflammation group (60% *vs.* 26%), while biological agents, i.e., rituximab and tocilizumab, showed better “drug survival” for Jo-1+ and PL-7+ ASyS patients with high inflammation, respectively, in our cohort.

**Conclusions:**

ASyS with recurrent systemic inflammatory episodes reflects a subtype of more aggressive and refractory disease in the spectrum of ASyS. Increased awareness of this subtype might lead to more appropriate management.

## Introduction

Anti-synthetase syndrome (ASyS) is one of the most common forms of idiopathic inflammatory myopathies (IIMs) in adults. Although ASyS shares many features with dermatomyositis (DM) and polymyositis, it has distinctive serological and clinical patterns (1). Serological hallmarks, i.e., anti-tRNA-synthetase antibodies (ARSs), have been identified, with anti-Jo-1 (histidyl-) ARS being the most common, followed by anti-PL-7 (threonyl-), anti-PL-12 (alanyl-), anti-EJ (glycyl-), and anti-OJ (isoleucyl-) antibodies, which are tested routinely in clinical practice. ASyS encompasses a cluster of clinical features, including myositis, interstitial lung disease (ILD), arthralgia or arthritis, Raynaud’s phenomenon, fever, and cutaneous manifestations such as mechanic’s hands and DM-like rashes, forming a constellation under the name ASyS (2). A meta-analysis including 27 idiopathic inflammatory myopathy studies (n=3,487) (3) found that patients positive for ARSs presented significantly more ILD (70%, CI 63–73), arthralgia (62%, CI 59–65), fever (43%, CI 43–47), and Raynaud’s phenomenon (47%, CI 43–51) than patients with other myositis-specific autoantibodies (mainly anti-Mi2 and anti-SRP) (4).

It is noteworthy that fever is a common and prominent clinical manifestation of ASyS, which is different from other IIMs, with a reported incidence varying from 26% to 61% (5, 6). Although most disease courses of ASyS are chronic with good-to-moderate response to conventional immunosuppressive treatments, patients who presented with recurrent episodes of fever and elevated acute phase reactants due to systemic inflammation were deemed to be more aggressive and difficult to treat.

In the current study, by analyzing a retrospective ASyS cohort, we focused on a subgroup of patients who presented with recurrent non-infectious fever and systemic inflammation. Our aim was to delineate the clinical characteristics of this special subtype in the spectrum of ASyS in a real-world setting.

## Methods

### Study Cohort

A retrospective cohort was established with consecutive patients diagnosed with ASyS referred to the Department of Rheumatology, Renji Hospital South Campus, Shanghai Jiao Tong University School of Medicine, from January 2013 to January 2020. The inclusion criteria were a clinical diagnosis of ASyS, with definitive serology findings of one of the five ARSs (Jo-1, PL-7, PL-12, EJ, and OJ) tested, along with at least one triad finding, encompassing myositis, arthritis, and ILD (1). The exclusion criteria were patients with malignancy within 3 years before or after the ASyS diagnosis and overlapping with other connective tissue diseases, such as systemic lupus erythematosus (SLE) and systemic sclerosis (SSc). Erosive or non-erosive arthritis with or without the presence of rheumatoid factor (RF) and anti-citrullinated protein antibody (ACPA) in ASyS patients had been demonstrated (7, 8). Thus, rheumatoid arthritis classification criteria were not implemented as exclusion criteria. This study protocol was approved by the internal ethics committee with informed consent for desensitized clinical data collection obtained from all patients.

Patient demographic information, clinical manifestations, laboratory tests, radiographic findings, and treatments were all retrospectively collected and evaluated. All eligible patients were ranked by the total number of systemic inflammation attacks.

### Terminologies

An *attack* of systemic inflammation was defined as acute episode of fever (with a documented temperature of 38°C or higher) during the disease course with elevated acute phase reactant (ESR >20 mm/h and/or CRP>8 mg/L), not otherwise explained, such as infection or drug fever, and was controlled only by enhanced immunosuppression (glucocorticoids and/or immunosuppressants). Recurrent fever within 1 month was only counted once.

*Fever at disease onset* referred to fever attack within 3 months from the onset of disease.

*Interstitial lung disease (ILD)* was identified by chest high-resolution CT (HRCT) with or without a consistent pulmonary function test. Radiological patterns of ILD were predominantly classified as usual interstitial pneumonia (UIP), non-specific interstitial pneumonia (NSIP), or organizing pneumonia (OP) according to the 2002 American Thoracic Society/European Respiratory Society classification criteria (9). All HRCT images were independently evaluated by two experienced investigators who were blinded to the clinical information.

*Rapid progressive ILD (RPILD)* including acute/subacute interstitial pneumonia was defined as the deterioration of the radiological interstitial changes with progressive dyspnea and hypoxemia associated with ILD within 3 months (10), which was attributed to ASyS *per se* rather than other causes such as infection, heart failure, or pulmonary embolism.

*Myositis* was defined as proximal muscle weakness and/or pain along with creatinine kinase elevation, with a compatible muscle magnetic resonance or electromyography or muscle biopsy findings.

*Refractory disease* was defined as exposure to at least three disease-modifying antirheumatic drugs (DMARDs), including methotrexate, azathioprine, cyclophosphamide, mycophenolate mofetil, cyclosporine, tacrolimus, leflunomide, and biological DMARDs (bDMARDs), namely, rituximab or tocilizumab, as the DMARDs used in our cohort, given sequentially or concomitantly.

*A good response* to a given DMARD was defined as clinical improvement without fever, active arthritis or myositis, or worsening pulmonary function test results and/or chest HRCT images and allowed glucocorticoids to be tapered to a maintenance prednisone dose of 5 to 10 mg per day or equivalent dosage (11); otherwise, the patient was categorized as a poor responder. *Undetermined response* was for patients still under follow-up and glucocorticoid tapering but not reaching a maintenance dosage.

### Detection of Myositis-Specific Autoantibodies

The identification of the anti-synthetase autoantibodies (anti-Jo-1, anti-PL-7, anti-PL-12, anti-OJ, anti-EJ) was determined by the Euroline Autoimmune Inflammatory Myopathies 16 Ag kit (Euroimmun, Luebeck, Germany). Simultaneously, a Bio-Plex Pro 2200 (Bio-Rad, USA) immunoassay system for Luminex-liquichip was used to detect autoantibodies against extractable nuclear antigens (ENA, anti-Jo1 included) and ACPA.

### Statistical Analysis

Categorical variables were compared using Fisher’s exact test or Pearson Chi-square test, while continuous variables were compared for two groups using independent sample Student’s t test or Mann-Whitney U test, as appropriate. One-way ANOVA or Kruskal–Wallis rank sum tests were performed for multiple comparisons. Multivariate logistic regression analysis was performed to assess the independent risk factors and presented as odds ratios [ORs with 95% confidence intervals (CIs)]. All analyses were performed using SPSS V.19 (Armonk, NY, USA) or GraphPad 5.0 (San Diego, CA, USA) software. The difference was considered statistically significant when the p-value was less than 0.05.

## Results

### Study Cohort

**Figure 1:**
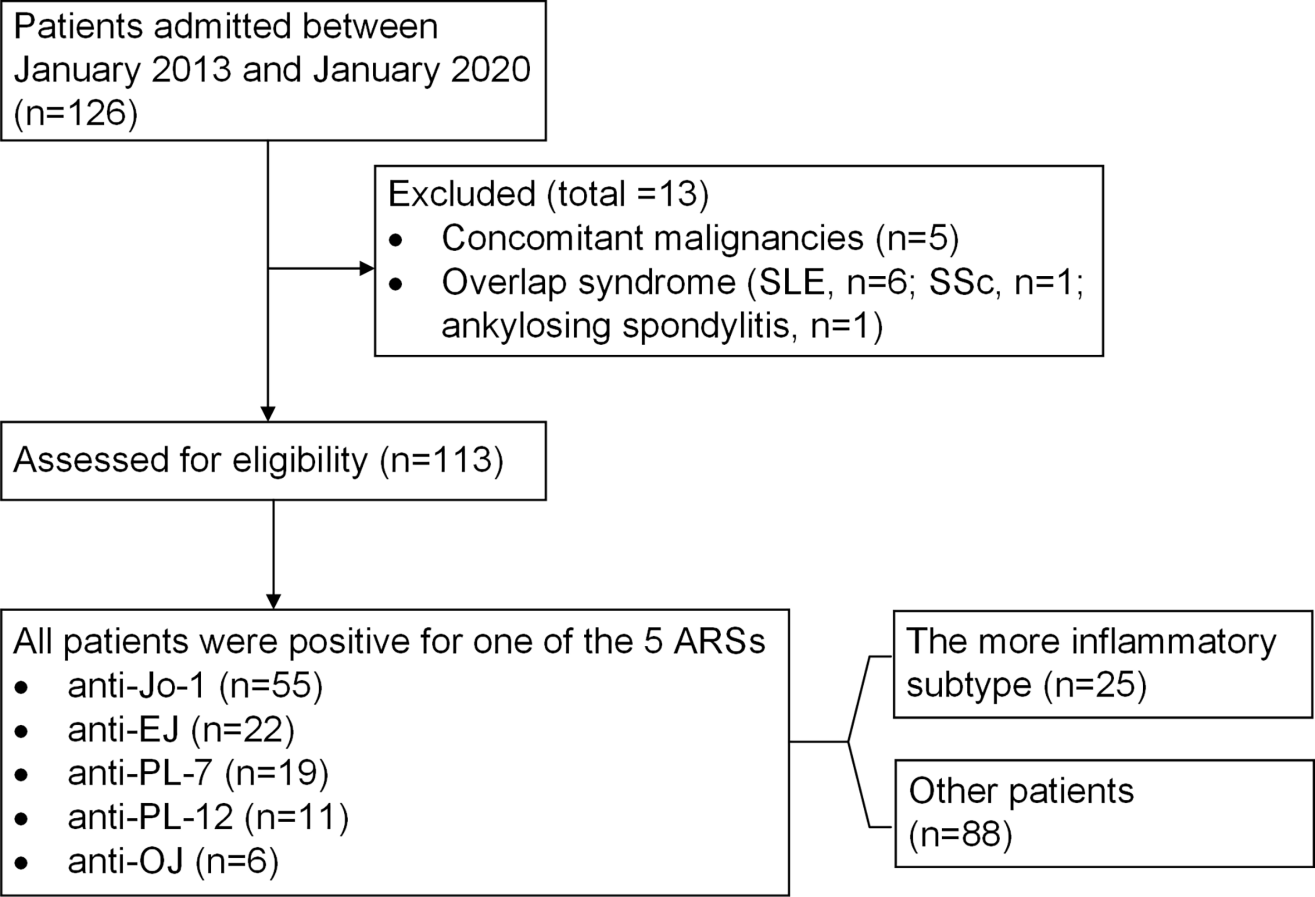
Flowchart of patients with ASyS.

We initially included 126 ASyS patients between January 2013 and January 2020 (**Figure 1**). Concomitant malignancies within 3 years (n=5, one pancreatic cancer, one lung cancer, one colon cancer, one breast cancer, and one for high clinical suspicion of cancer with bloody pericardial effusion/tamponade who deceased rapidly after being complicated with pulmonary embolism, for whom cytological or pathological evidence of malignancy was not established) and overlap syndrome (SLE, n=6; SSc, n=1; ankylosing spondylitis, n=1) were excluded. Ultimately, 113 patients were eligible, with 55 patients positive for anti-Jo1 (49%), 22 for anti-EJ (20%), 19 for anti-PL7 (17%), 11 for anti-PL12 (10%), and 6 for anti-OJ (5%). The average age at disease onset was 50 years, with a female predominance (76%), and the mean follow-up time was 58 ± 56 months. Myositis and ILD were present in 64% and 89% of patients, respectively. Seven patients died during follow-up and had a 6% 5-year mortality rate in this cohort. The profile is consistent with other ASyS cohorts reported (12).

### Fever Attacks

**Figure 2:**
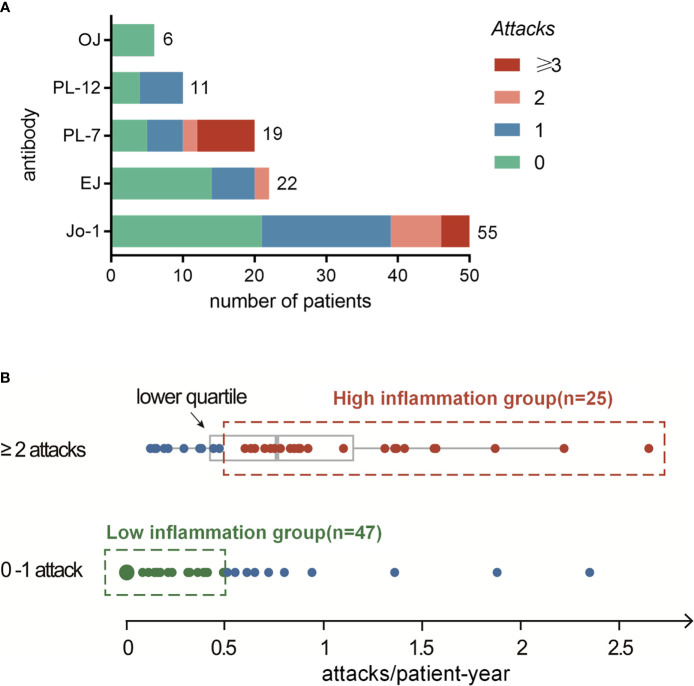
**(A)** Distribution analysis of systemic inflammation attacks in different ARS+ ASyS. **(B)** High-inflammation group is determined as the patients with ≥2 attacks and higher than 0.5 attacks per year during the follow up. Low-inflammation group is determined as patients with no more than one attack and a frequency <0.5 attack/patient-year.

Overall, 67 patients (59%) experienced fever attacks during the observed follow-up time; of those, 31 (27%) had one, 18 (16%) had two, and 18 (16%) had three or more attacks. As shown in **Table 1**, significant intergroup differences (according to the numbers of attacks) were revealed for fever at disease onset (p<0.001) and RPILD (p=0.002), which favored patients with two or more attacks. There were no significant intergroup differences in myositis, arthralgia, Raynaud’s phenomenon, mechanic’s hands, or DM-like rashes. The most common radiological pattern of ILD associated with ASyS was NSIP (73%); meanwhile, patients also presented OP (3%), UIP (4%), or NSIP/OP overlap radiological patterns (11%). Of the 12 patients described as a radiological NSIP/OP overlap pattern, up to eight patients presented as RPILD, which was in line with the predisposition of this NSIP/OP overlap pattern among patients with more attacks. Regarding ARSs, 53% of PL-7 patients presented two or more attacks. Stacked column plots displayed discordance of the distribution of such attacks in different ARS subtypes, with a higher cumulative number of attacks among Jo-1+ and PL-7+ ASyS patients (**Figure 2A**). No significant difference was observed in anti-Ro52, anti-Ro60, anti-La, ACPA, and anti-Pm-Scl. The treatment difference was prominent, with more DMARD (biologics included) exposure among patients with more attacks (p<0.001).

**Table 1:** Clinical manifestations regarding the numbers of attacks of systemic inflammation in an ASyS Chinese cohort.

	Attacks					
	Total cohort	0	1	2	≥3	*Global P value*
No. of patients	113	46 (41%)	31 (27%)	18 (16%)	18 (16%)	
Age at onset, years	50 ± 14	51 ± 13	51 ± 15	52 ± 14	47 ± 14	*0.632*
Female	86 (76%)	37 (80%)	20 (65%)	15 (83%)	14 (78%)	*0.378*
Disease duration, months	58 ± 56	51 ± 65	45 ± 34	74 ± 60	73 ± 33	*0.129*
Attacks per patient-year^*^	0.39 ± 0.54	0	0.43 ± 0.39	0.73 ± 0.72	1.00 ± 0.42	*0.000*
Fever at disease onset*	44 (39%)	0 (0%)	19 (61%)	12 (67%)	13 (72%)	0.000
***Clinical findings***						
Myositis	72 (64%)	26 (57%)	22 (71%)	10 (56%)	14 (78%)	*0.285*
Arthralgia	55 (49%)	17 (37%)	17 (55%)	11 (61%)	10 (56%)	*0.216*
ILD	102 (90%)	40 (87%)	28 (90%)	18 (100%)	16 (89%)	*0.466*
HRCT pattern of ILD						
NSIP	82 (73%)	35 (76%)	23 (74%)	10 (56%)	14 (78%)	*0.363*
OP	3 (3%)	1 (2%)	1 (3%)	1 (6%)	0 (0%)	*0.763*
NSIP/OP overlap^*^	12 (11%)	2 (4%)	3 (10%)	7 (39%)	0 (0%)	*0.000*
UIP	5 (4%)	2 (4%)	1 (3%)	0 (0%)	2 (11%)	*0.417*
RPILD^*^	17 (16%)	3 (7%)	4 (13%)	8 (44%)	2 (11%)	*0.002*
DM-like rashes	64 (57%)	24 (52%)	24 (77%)	8 (44%)	8 (44%)	*0.051*
Mechanic’s hand	27 (24%)	11 (24%)	10 (32%)	2 (11%)	4 (22%)	*0.431*
Raynaud’s phenomenon	15 (13%)	8 (17%)	3 (10%)	1 (6%)	3 (17%)	*0.611*
Serositis	42 (39%)	15 (36%)	10 (32%)	11 (65%)	6 (33%)	*0.124*
***Antibodies***						
Subtypes of ARS						
Anti-Jo-1	55 (49%)	20 (44%)	13 (42%)	12 (67%)	10 (56%)	*0.292*
Anti-EJ^*^	22 (20%)	14 (30%)	5 (16%)	3 (17%)	0 (0%)	*0.031*
Anti-PL-7*	19 (17%)	3 (7%)	6 (19%)	2 (11%)	8 (44%)	0.005
Anti-PL-12	11 (10%)	4 (9%)	6 (19%)	1 (6%)	0 (0%)	*0.159*
Anti-OJ	6 (5%)	5 (11%)	1 (3%)	0 (0%)	0 (0%)	*0.303*
Anti-Ro52	79 (73%)	34 (77%)	20 (67%)	13 (81%)	12 (67%)	*0.611*
Anti-Ro60	27 (25%)	9 (21%)	6 (19%)	4 (25%)	8 (47%)	*0.169*
Anti-La	14 (13%)	4 (9%)	2 (7%)	2 (11%)	6 (33%)	*0.060*
Anti-Pm-scl	6 (5%)	1 (2%)	1 (3%)	1 (6%)	3 (17%)	*0.111*
ACPA	12 (11%)	4 (9%)	2 (7%)	3 (17%)	3 (18%)	*0.523*
***Treatments***						
Maintenance GC, mg/d	10.2 ± 6.1	8.5 ± 5.1	7.4 ± 5.2	10.6 ± 5.8	11.0 ± 5.4	*0.062*
cDMARDs						
Methotrexate	24 (21%)	7 (15%)	6 (19%)	7 (39%)	4 (22%)	*0.219*
Azathioprine	52 (46%)	20 (44%)	13 (42%)	10 (56%)	9 (50%)	*0.777*
Cyclophosphamide	47 (42%)	17 (37%)	13 (42%)	8 (44%)	9 (50%)	*0.803*
Mycophenolate mofetil	45 (40%)	22 (48%)	9 (29%)	4 (22%)	10 (56%)	*0.074*
Cyclosporine	17 (15%)	6 (13%)	7 (23%)	1 (6%)	3 (17%)	*0.419*
Tacrolimus	21 (19%)	8 (17%)	4 (13%)	2 (11%)	7 (39%)	*0.099*
Leflunomide	4 (4%)	2 (4%)	0 (0%)	0 (0%)	2 (11%)	*0.179*
bDMARDs						
Rituximab^*^	28 (25%)	8 (17%)	6 (19%)	5 (28%)	9 (50%)	*0.045*
Tocilizumab^*^	5 (4%)	0 (0%)	1 (3%)	1 (6%)	3 (17%)	*0.004*
Cumulative DMARDs exposure ≥3^*^	34 (30%)	10 (22%)	5 (16%)	5 (28%)	14 (78%)	*0.000*
Use of bDMARDs^*^	33 (29%)	8 (17%)	7 (22%)	6 (33%)	12 (67%)	*0.003*
Deaths	7 (6%)	4 (9%)	2 (7%)	0 (0%)	1 (6%)	*0.430*

Data are presented as mean ± SD for continuous variables and number (frequency) for categorical variables. Missing data<5%.

*global p value < 0.05.

ILD, interstitial lung disease; RPILD, Rapidly Progressive Interstitial Lung Disease; DM, dermatomyositis; ARS, aminoacyl tRNA synthetase; ACPA, anti-citrullinated protein antibody; GC, glucocorticoids, expressed as the daily dose of prednisone equivalent; cDMARDs, Conventional disease modifying antirheumatic drugs; bDMARDs, Biologic disease-modifying antirheumatic drugs; DMARDs, disease-modifying antirheumatic drugs (including cDMARDs and bDMARDs).

### Definition and Characterization of High-Inflammation Subtype in ASyS Patients

We next calculated the frequency of attacks (per patient-year) for all eligible patients. The mean frequency of such attacks in our cohort was 0.39 per patient-year. For the ASyS patients manifested by recurrent (two or more) attacks (n=36), the median frequency was 0.77 (IQR 0.40, 1.26) attacks/patient-year. The lower quartile rounding to 0.5 attack/patient-year was set as the cutoff, and patients with higher frequency attacks and had at least two attacks were defined as high-inflammation group (n=25). For comparison, low-inflammation group was defined as patients who had only 0–1 attacks and no additional attack after at least 2 years of follow-up (<0.5 attack/patient-year, n=47) (**Figure 2B**). Of note, the rest of the patients who met neither definition were categorized as undetermined (n=41). Comparison analyses between undetermined *vs.* high-inflammation group and low-inflammation group were presented as **Supplementary Table 1**. The attack frequencies in the high-inflammation and low-inflammation groups were 1.12 ± 0.53 and 0.07 ± 0.13 (attacks/patient-year), respectively.

Univariate analysis comparing high-inflammation group *versus* low-inflammation group suggested that the more inflammatory subtype of ASyS patients was more likely to have fever and RPILD as the first presentation (84% *vs.* 21%, p<0.001 and 40% *vs.* 9%, p=0.003, respectively, both p<0.01). Importantly, anti-PL-7 (p=0.014) was significantly correlated with high-inflammation subtype compared to other ARSs. These three baseline parameters were subjected to multivariate logistic regression. Finally, fever at disease onset and RPILD remained independent risk factors for the high-inflammation group (p<0.001 and p=0.016, respectively) (**Table 2**). To further characterize the high-inflammation subtype, the 25 patients were divided into anti-Jo-1+ and Anti-PL-7+ ASyS patients (**Table 3**). Anti-PL-7+ ASyS patients with high inflammation showed higher CRP/ESR/Ferritin/IL-6 levels during attack compared with Anti-Jo-1+ ASyS patients, but only ESR reached the statistical significance (p<0.01). To evaluate the hyper-inflammation status, HScore were calculated (13), and the median HScore of high-inflammation subtype was around 70 (the probability of having Macrophage Activation Syndrome (MAS) <1%). In addition, PL-7+ ASyS patients with high inflammation had a higher positive rate of ACPA (p=0.028), although no difference in terms of the presence of arthralgia/arthritis compared to Jo-1+ ASyS patients.

**Table 2:** Univariate and multivariate analyses in ASyS patients with high inflammation and low inflammation.

	Low-inflammation group (n=47)	High-inflammation group (n=25)	*P-value*	Multivariate analysis		
				OR	95% CI	*P-value*
***Baseline characteristics***						
Age, years	50 ± 14	49 ± 15	*0.923*			
Gender, female	38 (81%)	19 (76%)	*0.629*			
Fever at disease onset	10 (21%)	21 (84%)	0.000^*^	20.62	5.05, 84.14	0.000^*^
RPILD	4 (9%)	10 (40%)	0.003^*^	8.03	1.48, 43.52	0.016^*^
Anti-Jo-1	20 (43%)	16 (64%)	*0.083*			
Anti-PL-7	5 (11%)	9 (36%)	0.014^*^			
***Follow-up characteristics***						
Attacks per patient-year	0.07 ± 0.13	1.12 ± 0.53	0.000^*^			
Maintenance GC, mg/d	8.5 ± 5.3	11.1 ± 6.1	*0.069*			
Cumulative DMARDs exposure≥3	12 (26%)	15 (60%)	0.004^*^			
Use of bDMARDs	9 (19%)	18 (72%)	0.000*			
Deaths	3 (6%)^a^	1 (4%)^b^	*1.000*			

Data are presented as mean ± SD for continuous variables and number (frequency) for categorical variables.

OR, Odds Ratio, ^*^p < 0.05.

a Two patients died from opportunistic infection. Another death was family members reported with definitive cause of death unavailable.

b The only patient in high-inflammation group died from gastrointestinal (GI) bleeding.

**Table 3:** Characterization of the high-inflammation group in ASyS patients (Anti-Jo-1+ *vs.* Anti-PL-7+).

	High-inflammation group (n=25)		P value
	Anti-Jo-1 (n=16)	Anti-PL-7 (n=9)	
Age at onset, years	51 ± 16	46 ± 13	*0.462*
Female	12 (75%)	7 (78%)	*1.000*
Disease duration, months	48 ± 35	63 ± 28	*0.283*
Fever at disease onset	13 (81%)	8 (89%)	*1.000*
Attacks per patient-year	1.14 ± 0.56	1.09 ± 0.50	*0.820*
***Clinical findings***			
Myositis	11 (69%)	4 (44%)	*0.397*
Arthralgia	11 (69%)	5 (56%)	*0.671*
ILD	14 (88%)	9 (100.0%)	*0.520*
RPILD	6 (38%)	3 (33%)	*1.000*
DM-like rashes	5 (31%)	6 (67%)	*0.115*
Mechanic’s hand	2 (13%)	3 (33%)	*0.312*
Raynaud’s phenomenon	1 (6%)	1 (11%)	*1.000*
***Laboratory values***			
Anti-Ro52	12 (75%)	6 (67%)	*0.635*
Anti-Ro60	4 (25%)	5 (56%)	*0.383*
Anti-La	4 (25%)	2 (22%)	*1.000*
ACPA^†^	1 (6%)	4 (50%)	*0.028*
CRP*, mg/L	24.1 ± 26.5	33.6 ± 17.3	*0.346*
ESR*, mm/h	19.6 ± 11.6	55.1 ± 27.7	*0.002*
Ferritin*, ng/ml	300.6 ± 219.5	438.9 ± 532.7	*0.910*
IL-6*^†^, pg/ml	6.5 ± 6.8	9.8 ± 9.9	*0.365*
HScore*	68 ± 30	72 ± 30	*0.778*
***Treatments***			
Maintenance GC, mg/d	12.6 ± 6.4	8.5 ± 4.8	*0.107*
Cumulative DMARDs exposure ≥3	8 (50%)	6 (67%)	*0.691*
Use of bDMARDs	11 (69%)	7 (78%)	*1.000*
Response rate of RTX	9/11 (82%)	1/3 (33%)	*0.176*
Response rate of TCZ	–	4/4 (100%)	*-*

Data are presented as mean ± SD for continuous variables and number (frequency) for categorical variables.

*Collected or calculated during the fever attack phase.

^†^Valid cases for variables with missing data: ACPA (n= 24), IL-6 (n=21).

### Response to Treatment

**Figure 3:**
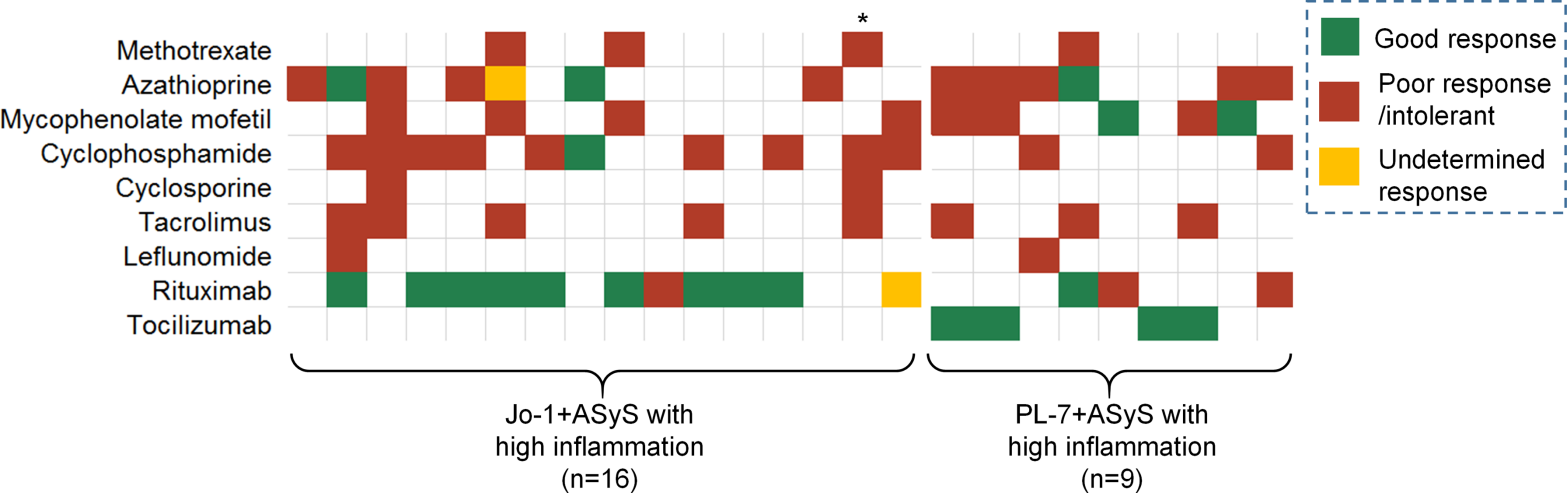
Treatment response in the high-inflammation group of ASyS patients. Each column represents one individual patient. * Deceased due to GI bleeding.

The more inflammatory subtype of ASyS patients was more refractory to treatments, as evidenced by higher (>=3) cumulative DMARD exposures (60% *vs.* 26%) and more bDMARD exposures (72% *vs.* 19%). The responsiveness to specific DMARDs is presented by a heat map with green, red, and yellow colors, representing good, poor, and undetermined responses, respectively (**Figure 3**). The overall responsiveness to conventional DMARDs among ASyS patients with high inflammation was poor. Notably, among 11 out of the 16 Jo-1+ ASyS patients with high inflammation receiving rituximab, nine patients had a good response. Conversely, among three out of nine PL-7+ ASyS patients with high inflammation treated with rituximab, two failed treatment; whereas tocilizumab exposure (intravenous standard dose at 8 mg/kg every 4 weeks) in another four PL-7+ ASyS patients achieved a good response.

## Discussion

Currently, there is no international consensus for the classification of ASyS. However, according to the largest ASyS cohort AENEAS (American and European NEtwork of Antisynthetase Syndrome) (1), a combination of ARS and any manifestation of the “triad” (myositis, ILD, and arthritis) might be a legitimate ASyS. By using our single-center cohort, we focused on fever attacks beyond the classic triad among Chinese ASyS patients. We have found that ~60% of patients experienced one or more febrile attacks during an average 5-year follow-up. In addition, ~30% of the patients had recurrent attacks (>=2). We took those with more than two febrile attacks and combined with the time-adjusted frequency of attacks to further define this subpopulation.

These patients, represented ~20% of our ASyS cohort and had three distinctive features. First, these patients experienced more RPILD (40%) and tended to present fever (84%) within 3 months from disease onset, providing important clues for early recognition of the more inflammatory phenotype. Indeed, RPILD has been reported in 7.8–29.2% of ASyS patients (14). The link between recurrent fever attacks and RPILD suggests that RPILD may be a component of profound systemic inflammation. It is noteworthy that this hyper-inflammation status in ASyS is quite different from the classic macrophage activation syndrome, according to our data.

Second, the correlation between PL-7 and the more inflammatory phenotype was informative. The ARS serology of high-inflammation subtype in our cohort was exclusively either PL-7+ or Jo-1+. Nearly ~50% of PL-7 patients in our cohort were labeled as high-inflammation subtype, which might “ring-a-bell” for clinical judgment when PL-7 was encountered. On the other hand, ~30% of Jo-1 patients were classified as high-inflammation group. Therefore, the number of Jo-1+ ASyS patients with high inflammation was more attributed to the high prevalence of Jo-1 in the ASyS cohort. Likewise, none of the EJ+, PL-12, or OJ+ ASyS patients in our cohort fulfilled the high-inflammation subtype definition, which might simply be because of the small number of patients. As an example, in a larger single-center Chinese ASyS cohort (n=234), the authors reported 46 EJ+ patients who had similar occurrences of fever (60.9%) and RPILD (21.7%) (6). Some patients might well be labeled as high-inflammation subtype. And there is no explicit relationship between high-inflammation phenotype and the ARS antibodies that are not routinely measured.

Third, high-inflammation subtype of ASyS patients was more refractory to conventional immunosuppressive therapies. In this descriptive study, we observed a good response to rituximab for most Jo-1+ ASyS patients with high inflammation. The rituximab in myositis (RIM) study and subanalysis suggested that Jo-1+ patients were better responders to rituximab (15, 16). The observation in our study probably reflects evidence-driven decision-making processes. More interestingly, for PL-7+ ASyS patients with high inflammation, this is the first anecdotal report of a promising response to tocilizumab. As an interleukin-6-receptor inhibitor, tocilizumab has been extensively tried out in various hyperinflammatory status. The underlying physiopathology in high-inflammation subtype of ASyS patients might turn out to be a good rationale to justify using tocilizumab instead of conventional treatment. Subsequent clinical trials in PL-7+ ASyS patients are needed to fully address its possible efficacy.

The disease spectrum of ASyS is likely to be continuum, and the high-inflammation subtype of ASyS definition we used in the current study is somewhat arbitrary; however, the terminology reflects the endeavor to understand the systemic inflammatory aspect of ASyS. Another major limitation of this study is the retrospective single-center design and limited number of patients. Larger, independent, multicenter studies ideally covering different ethnic populations are mandatory to thoroughly evaluate this intriguing subtype of ASyS. This will be the only way to surpass the possible “auto-analysis” issue. Finally, the treatment options in the current study were highly investigator-dependent; nevertheless, the data might reflect “drug survival” during “trial-and-error” selection in a real-world setting.

## Data Availability Statement

The raw data supporting the conclusions of this article will be made available by the authors, without undue reservation.

## Ethics Statement

The studies involving human participants were reviewed and approved by Renji Hospital, Shanghai Jiaotong University School of Medicine. The patients/participants provided their written informed consent to participate in this study. Written informed consent was obtained from the individual(s) for the publication of any potentially identifiable images or data included in this article.

## Author Contributions

Study conception and design: SY, SS. Acquisition of data: SS, ZC, DZ, LG, FS, WW, WX, JC, and JL. Analysis and interpretation of data: SY, SS, DZ, XW, TL. Drafting the article: SS and DZ. Revising the article: SY, ZC, XW, and TL. All authors contributed to the article and approved the submitted version.

## Conflict of Interest

The authors declare that the research was conducted in the absence of any commercial or financial relationships that could be construed as a potential conflict of interest.

## Publisher’s Note

All claims expressed in this article are solely those of the authors and do not necessarily represent those of their affiliated organizations, or those of the publisher, the editors and the reviewers. Any product that may be evaluated in this article, or claim that may be made by its manufacturer, is not guaranteed or endorsed by the publisher.
