# Supplementary material for: Description and Analysis of a Novel Subtype of the Anti-Synthetase Syndrome Characterized by Frequent Attacks of Fever and Systemic Inflammation in a Single-Center Cohort Study
Source: Front Immunol. 2021 Sep 23;12:729602. doi: 10.3389/fimmu.2021.729602 (PMC8495196; doi:10.3389/fimmu.2021.729602)
Supplement: Supplementary file 1 [file Table_1.pdf]

Supplementary Table 1. Comparison analyses between undetermined vs high-inflammation group and low-inflammation group

	Undetermined	High-inflammation group	Low-inflammation group	*P value* [a]	*P value* [b]
No. of patients	41	25	47	-	-
Age at onset, years	52±14	49±15	50±14	0.331	0.281
Female	29(71%)	19(76%)	38(81%)	0.641	0.267
Disease duration, months	51±52	53±33	64±63	0.142	0.006
Attacks per patient-year	0.32±0.39	1.12±0.53	0.07±0.13	0.000	0.000
Fever at disease onset	13(32%)	21(84%)	10(21%)	0.000	0.267
Clinical findings					
Myositis	28(68%)	15(60%)	29(62%)	0.493	0.519
Arthralgia	19(46%)	16(64%)	20(43%)	0.163	0.721
ILD	38(93%)	23(92%)	41(87%)	1.000	0.494
HRCT pattern of ILD					
NSIP	32(78%)	15(60%)	35(75%)	0.116	0.694
OP	1(2%)	1(4%)	1(2%)	1.000	1.000
NSIP/OP overlap	3(7%)	6(24%)	3(6%)	0.072	1.000
UIP	2(5%)	1(4%)	2(4%)	1.000	1.000
RPILD	4(10%)	9(36%)	4(9%)	0.023	1.000
DM-like rashes	26(63%)	11(44%)	27(57%)	0.123	0.568
Mechanic's hand	9(22%)	5(20%)	13(28%)	0.851	0.537
Raynaud's phenomenon	7(17%)	2(8%)	6(13%)	0.464	0.570
Serositis	10(26%)	12(48%)	20(46%)	0.066	0.061

Antibodies					
Subtypes of ARS					
Anti-Jo-1	19(46%)	16(64%)	20(43%)	0.163	0.721
Anti-EJ	7(17%)	0(0%)	15(32%)	0.039	0.109
Anti-PL-7	5(12%)	9(36%)	5(11%)	0.022	1.000
Anti-PL-12	7(17%)	0(0%)	4(9%)	0.039	0.226
Anti-OJ	3(7%)	0(0%)	3(6%)	0.283	1.000
Anti-Ro52	31(82%)	18(75%)	30(65%)	0.535	0.094
Anti-Ro60	10(25%)	9(39%)	8(18%)	0.239	0.416
Anti-La	3(8%)	6(24%)	5(11%)	0.076	0.717
Anti-Pm-scl	2(5%)	3(12%)	1(2%)	0.359	0.596
ACPA	2(5%)	5(21%)	5(11%)	0.095	0.445
Treatments					
Maintenance pred, mg/d	8.1±4.7	11.1±6.1	8.5±5.3	0.069	0.970
Cumulative DMARDs exposure ≥3	7(17%)	15(60%)	12(26%)	0.000	0.336
Use of bDMARDs	8(20%)	18(72%)	9(19%)	0.000	0.966
Deaths	3(7%)	1(4%)	3(6%)	1.000	1.000

Data are presented as mean±SD for continuous variables and number (frequency) for categorical variables. Missing data＜5%

[a] High inflammation group vs Undetermined

[b] Low inflammation group vs Undetermined
